# Transcriptome profiling of mouse brains with *qkI*-deficient oligodendrocytes reveals major alternative splicing defects including self-splicing

**DOI:** 10.1038/s41598-017-06211-1

**Published:** 2017-08-08

**Authors:** Lama Darbelli, Karine Choquet, Stéphane Richard, Claudia L. Kleinman

**Affiliations:** 1 Segal Cancer Centre, Lady Davis Institute for Medical Research, Sir Mortimer B. Davis Jewish General Hospital, Montréal, Québec Canada; 2 Bloomfield Center for Research on Aging, Lady Davis Institute for Medical Research, Sir Mortimer B. Davis Jewish General Hospital, Montréal, Québec Canada; 3 Departments of Oncology and Medicine, McGill University, Montréal, Québec Canada; 4 Department of Human Genetics, McGill University, Montréal, Québec Canada

## Abstract

The *qkI* gene encodes a family of RNA binding proteins alternatively spliced at its 3′ end, giving rise to three major spliced isoforms: QKI-5, QKI-6 and QKI-7. Their expression is tightly regulated during brain development with nuclear QKI-5 being the most abundant during embryogenesis followed by QKI-6 and QKI-7 that peak during myelination. Previously, we generated a mouse conditional *qkI* allele where exon 2 is excised using *Olig2-Cre* resulting in QKI-deficient oligodendrocytes (OLs). These mice have dysmyelination and die at the third post-natal week. Herein, we performed a transcriptomic analysis of P14 mouse brains of QKI-proficient (*QKI* ^*FL*/*FL*;*-*^) and QKI-deficient (*QKI* ^*FL*/*FL*;*Olig2*-*Cre*^) OLs. QKI deficiency results in major global changes of gene expression and RNA processing with >1,800 differentially expressed genes with the top categories being axon ensheathment and myelination. Specific downregulated genes included major myelin proteins, suggesting that the QKI proteins are key regulators of RNA metabolism in OLs. We also identify 810 alternatively spliced genes including known QKI targets, *MBP* and *Nfasc*. Interestingly, we observe in *QKI* ^*FL*/*FL*;*Olig2*-*Cre*^ a switch in exon 2-deficient *qkI* mRNAs favoring the expression of the *qkI-5* rather than the *qkI-6* and *qkI-7*. These findings define QKI as regulators of alternative splicing in OLs including self-splicing.

## Introduction

The quaking (*qkI*) gene encodes a family of alternatively spliced isoforms of RNA binding proteins that function as master regulators of cell differentiation^1^. The deletion of these QKI isoforms in neural progenitors, muscles and monocytes causes severe differentiation defects^2–4^. A mouse conditional null allele of *qkI* has been generated by deleting exon 2 encoding part of the KH domain^2^. The deletion of the QKI isoforms in OLs using *Olig2-Cre* (*QKI* ^*FL*/*FL*;*Olig2*-*Cre*^) leads to severe CNS hypomyelination with tremors by post-natal day 10 (P10) and death at the third post-natal week. Ablation of *qkI* in adult mice using *PLP-CreERT* results in hindlimb paralysis, thoracic kyphosis and immobility by the third to fourth week post-4-hydroxytamoxifen (OHT) administration^2^. This work defines the QKI isoforms as major regulators of OL differentiation and maintenance. QKI also plays a pivotal role in monocytes, as its deletion using RNA interference blocked their differentiation into macrophages^4^. Moreover, a *qkI* haploinsufficient patient^5^ also exhibits monocyte to macrophage differentiation defects^4^. Indeed, in humans, the essential role of QKI is evidenced by a marked intolerance to loss-of-function (LoF) genetic variants: an analysis of protein-coding genetic variation in >60,000 human exomes^6^ shows that LoF variants are observed 14 times less frequently than expected by chance, with an associated probability of LoF intolerance of 0.96.

The *qkI* gene encodes KH-type RNA binding proteins that generates 3 major alternatively spliced mRNAs (5, 6, and 7 kb) encoding QKI-5, -6, and -7 that differ in their C-terminal 30 amino acids^7^. QKI-5 contains a nuclear localization signal in its unique C-terminal sequence and is exclusively nuclear^8^. Thus QKI-5 participates in nuclear roles such as in the regulation of alternative splicing. QKI-6 is distributed throughout the cell and forms heterodimers with QKI-5 and may perform both nuclear and cytoplasmic functions^9^. QKI-7 is predominantly cytoplasmic and both QKI-6 and QKI-7 are thought to function in mRNA export, mRNA stability and protein synthesis^10–15^. During embryonic brain development, QKI-5 isoform is the most abundant and its expression declines after the first two post-natal weeks, while the QKI-6 and QKI-7 isoforms are expressed later in embryogenesis and peak during myelination at the third post-natal week^7, 16^. What regulates the *qkI* gene and its alternative splicing is unknown.

The QKI isoforms all share identical KH domains flanked by QUA1 and QUA2 sequences that mediate sequence-specific recognition of an RNA element termed the QKI response element (QRE) with the following sequence ACUAAY-(N_1–20_)-UAAY^17^. Transcriptome-wide CLIP (crosslinking and immunoprecipitation) demonstrate that the QKI isoforms *in cellulo* bind the QREs and also showed that QKI associates predominantly with intronic sequences implying that alternative splicing regulation is one of its main nuclear functions^18^. The QKI isoforms were shown to regulate pre-mRNA splicing of myelin components^19^, and later this function of QKI was shown not to be restricted to brain, as QKI-5 also fulfills this role in vascular smooth muscle cells, monocytes and skeletal muscle^3, 4, 20^.

Herein, we report a transcriptomic analysis by RNA-Seq of entire brains of *QKI* ^*FL*/*FL*;*Olig2*-*Cre*^ mice compared to *QKI* ^*FL*/*FL*;-^ mice. The results are consistent with the hypomyelination phenotype of the mice and show that mRNAs encoding myelin components are severely downregulated^2^. We also identify alternative splicing defects especially in genes encoding myelin components. Interestingly, we noted that the absence of the QKI proteins also altered the alternative splicing of the *qkI* gene. Deletion of the *qkI* exon 2 generates a null allele, but switches the production of the *qkI* ^*Δexon2*^ mRNAs from being mainly *qkI-6* and *qkI-7* in wild type P14 brains to *qkI-5* in the brains of *QKI* ^*FL*/*FL*;*Olig2*-*Cre*^ mice. This work defines a self-splicing mechanism by which the *qkI* gene autoregulates its own alternative splicing in oligodendrocytes.

## Results

### QKI-depleted oligodendrocytes downregulate genes involved in myelination

**Figure 1:**
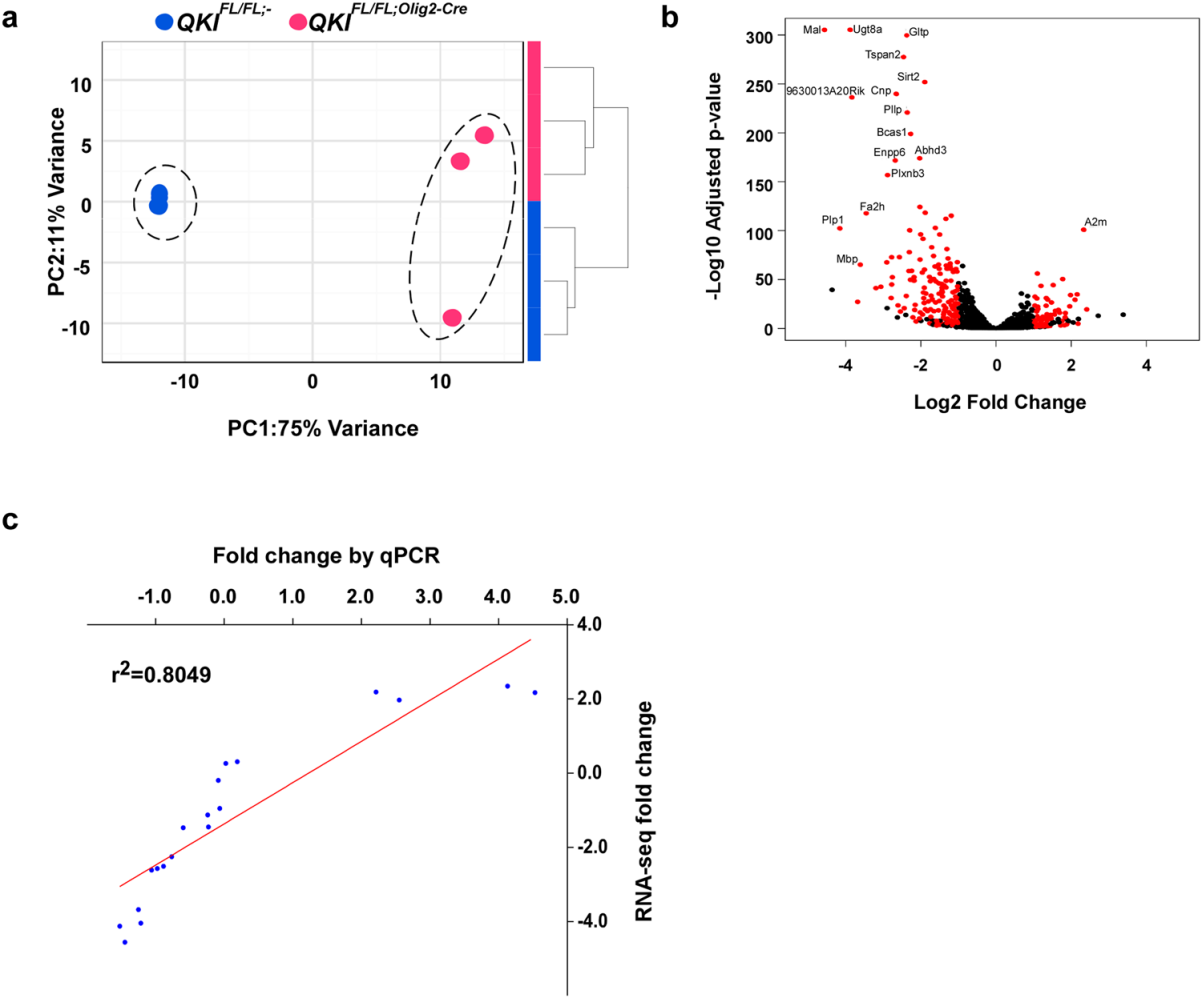
Gene expression profiles comparing brains of *QKI* ^*FL*/*FL*;*Olig2*-*Cre*^ *and QKI* ^*FL*/*FL*;-^ mice. (**a**) Global effects of ﻿*QKI* ^*FL/FL;Olig2-Cre*﻿^ on cells were evaluated by unsupervised clustering of samples based on expression profiles of 1,000 most variant genes. *QKI* ^*FL/FL;Olig2-Cre*^ and *QKI* ^*FL/FL;-*^ mice form robust, distinct clusters, both with hierarchical clustering (right) and Principal Component Analysis (left). (**b**) Volcano plot representing the results of differential expression analysis. Genes with adjusted p-value < 0.05, absolute fold change >2 and base mean >100 are colored in red. (**c**) Validation of RNA-seq data by qPCR with 19 transcripts. RNA-seq data strongly correlated with qPCR data (r^2^ = 0.8049, *p* < 0.0001, Pearson correlation coefficient).

**Figure 2:**
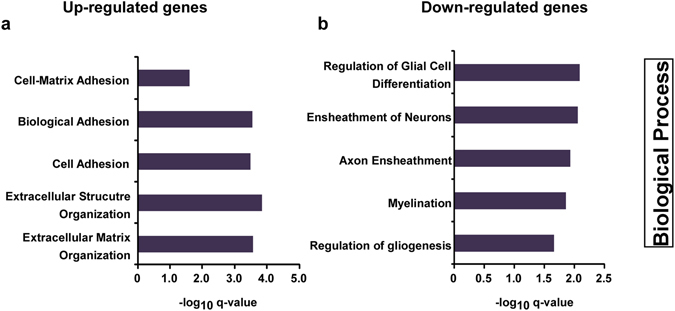
Gene ontology analysis. Gene Ontology analysis shows enriched Biological Processes in the lists of upregulated genes (a) and downregulated genes (b) in *QKI* ^*FL/FL;Olig2-Cre*^ mice. Genes with adjusted p-value < 0.05, absolute fold change >2 and a base mean >100 were used for the analysis.

To define the genome-wide alterations in gene expression and alternative splicing patterns in the absence of the QKI proteins, we isolated RNA from brains of *QKI* ^*FL*/*FL*;-^ and *QKI* ^*FL*/*FL*;*Olig2*-*Cre*^ mice (n = 3 females/genotype) and performed RNA-sequencing at an average of 133 million reads/sample (Supplementary Table S1). Sequencing and quality control metrics showed high quality reads, with a mean alignment rate greater than 90%. More than 95% of reads mapped to genes and there was low ribosomal and mitochondrial gene content (Supplementary Table S1). We first assessed the global impact of oligodendrocyte (OL)-specific depletion of the QKI RNA binding proteins by unsupervised clustering analysis of samples based on expression profiles. *QKI* ^*FL*/*FL*;-^ and *QKI* ^*FL*/*FL*;*Olig2*-*Cre*^ mice consistently formed distinct, robust clusters (Fig. 1a). Indeed, Principal Component Analysis (PCA) based on gene expression data showed that the first component (PC1) clearly separates *QKI* ^*FL*/*FL*;-^ and *QKI* ^*FL*/*FL*;*Olig2*-*Cre*^ mice, and explained 75% of the variance (Fig. 1a). Bootstrapped hierarchical clustering also separated the two groups with high bootstrap values (Supplementary Fig. S1a). The clustering is very robust, and is not affected by the number of genes or the algorithm used (Supplementary Fig. S1b). Taken together, these results indicate that QKI-depletion in oligodendrocytes induces major changes in gene expression in the brain. In fact, differential expression analysis showed statistically significant differences in 1,899 genes, representing 10.55% of expressed genes (n = 18,004). Of these, 229 had an absolute fold change greater than 2 (Fig. 1b and Supplementary Table S2). A high proportion (70%) of the significant genes is downregulated, in line with a scenario where a well-defined population of cells is depleted in the brain samples. Quantitative RT-PCR analysis of 19 genes (Supplementary Fig. S1c) showed strong correlation between RNA-seq fold change and RT-qPCR data (r^2^ = 0.8049, Fig. 1c). Gene Ontology (GO) analysis using the conservative list of 229 differentially expressed genes (with absolute fold change greater than 2) suggests that the downregulated biological processes are enriched for genes implicated in myelination and ensheathment of neurons (Fig. 2a,b), consistent with the hypomyelination phenotype of these mice^2^. The upregulated genes, on the other hand, are enriched for biological processes involving extracellular matrix organization and cell adhesion, and include a number of genes related to neuronal biology, once again reflecting the loss of oligodendrocyte populations in QKI-depleted mice and increase in proportion of cell populations of neuronal origin (Fig. 2a,b). Examples include genes related to axon guidance, such as Ntn4, or genes associated with neurological phenotypes, such as Col5a3 (Supplementary Table S2).

Loss of QKI did not lead to major changes in transcript levels of genes involved in oligodendrocyte lineage specification and precursor proliferation, including *Olig2*, *Nkx6*.*1*, *Id2*, *Id4*, and *Sox5*/*6* (Supplementary Table S2), consistent with maintenance of PDGFRα^+^ OLs in *QKI* ^*FL*/*FL*;*Olig2*-*Cre*^ mice^2^. QKI did appear to regulate RNA metabolism of transcription factors involved in OL differentiation and expression of myelin genes, including *Sox10*, *Myrf*, or myelin genes, including *Mag*, *Plp*, *Mbp*, *Mobp*, and *Sirt2* (Fig. 1c), consistent with the hypomyelination phenotype of the mice. The sequencing of the entire brain would reveal secondary events occurring, for example in neurons, as it is well-known that OLs tightly regulate the contacts and metabolism of neurons^21^. However, genes involved in neuronal specification and differentiation, including Neurogenin proneural genes, Achaete scute-like 1, as well as neurogenic differentiation^22^, were not affected by the loss of QKI. Therefore, loss of QKI in OLs exerts changes in gene expression that are specific to OL lineage transcripts.

### Alternative splicing changes observed in *QKI*^*FL*/*FL*;*Olig2*-*Cre*^ mice

**Figure 3:**
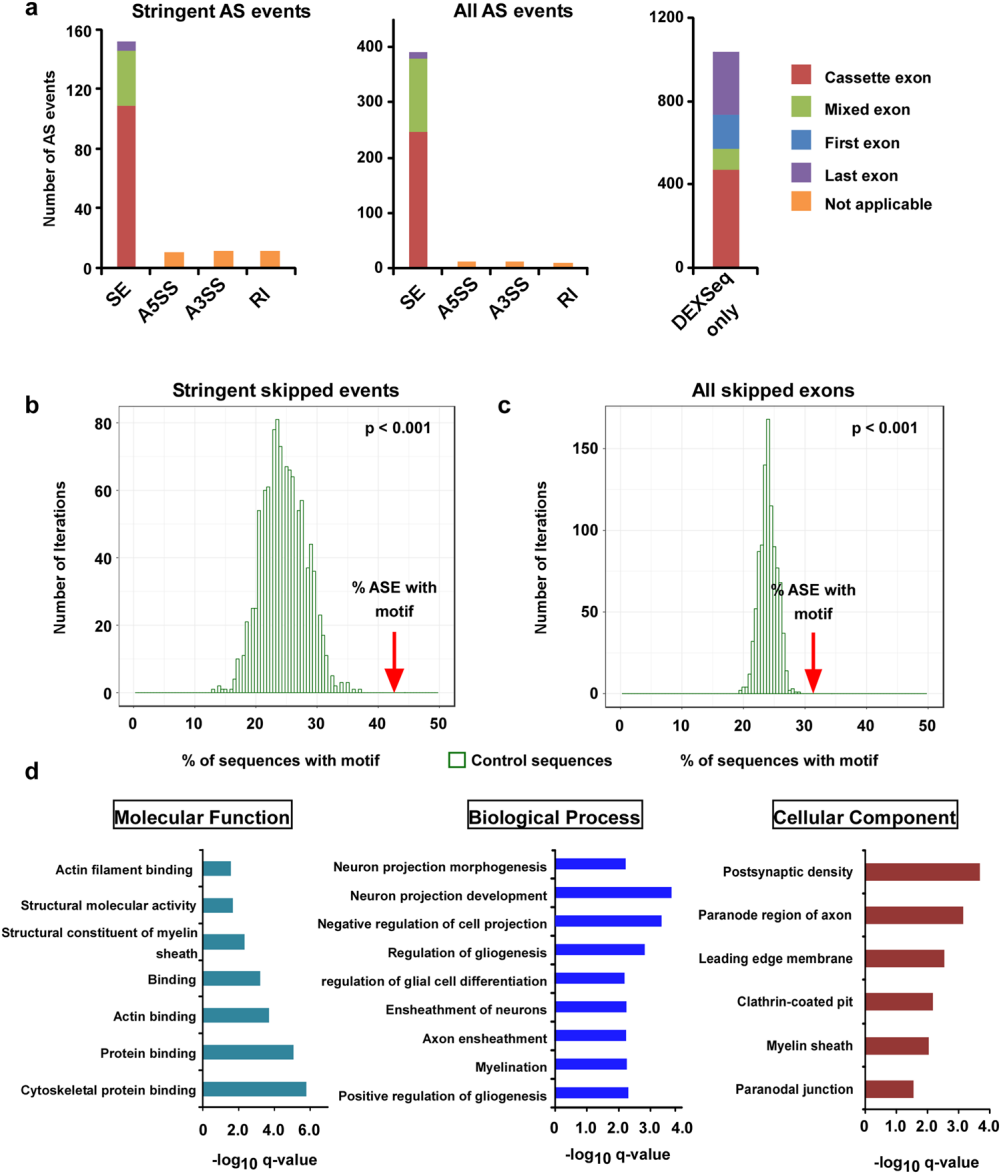
Brain alternative splicing patterns of mice with QKI-deficient oligodendrocytes. (**a**) Types of alternative splicing events identified in *QKI* ^*FL*/*FL*;*Olig2*-*Cre*^ mice. For skipped exons (SE), the type of exon affected is also indicated. Exons were considered to be “cassette exons” if they were not the first or last exon in any of the gene isoforms or “mixed exons” if they were not the first or last exon in at least one isoform. Abbreviations: SE: skipped exon, A5SS: alternative 5′ splice site, A3SS: alternative 3′ splice site, RI: retained intron. (**b**,**c**) Motif enrichment analysis. Percentage of sequences with the motif ACUAA in introns neighboring SE events compared to a set of background sequences for the stringent SE candidates called by both methods (b) or for all SE candidates (**c**). The occurrence of the motif was counted in a random set of non-alternatively spliced control sequences of the same size of the set of SE events. This was repeated 1,000 times to get the distribution of the motif occurrence in control sequences. The p-value corresponds to the empirical p-value of the enrichment for the set of SE events compared to the control distribution. (d) Gene Ontology analysis shows enriched molecular function, biological processes and cellular component in the list of genes where the stringent alternative splicing events were found. For Molecular Function, all GO terms with q-value < 0.01 are represented. For Biological Process and Cellular Component, GO terms with q-value < 0.01 and enrichment >5 are represented.

A previous search of alternative splicing events (ASE) within a defined subset of genes allowed us to identify 31 QKI-regulated ASEs in oligodendrocytes^2^. Here, we extended the search genome-wide to identify novel events and to assess the extent of AS regulation by QKI, using two different, complementary methods to infer splicing information from RNA-Seq data (DEXSeq and rMATs)^23, 24^. Genome-wide loss of QKI resulted in widespread AS changes, with 1,438 ASEs detected in 810 genes (Supplementary Tables S3–S5). Of the 31 identified with the candidate approach, 19 events were confirmed by RNA-seq including ASEs of *Nfasc*, *Magi*, *Lhfpl3*, *Mpzl1*, *Cadm1*, *Bin1*, *Vldlr*, *Plp1*, *Phka1*, *Mbp*, *Mobp*, *Grm5*, *Nhsl1*, and *Sirt2* (Supplementary Table S6). These data indicate that our RNA-seq analysis identified *bona fide* QKI RNA targets and that the majority of AS exons are novel events that have not been previously reported in *QKI* ^*FL*/*FL*;*Olig2*-*Cre*^ mice. A stringent list of ASEs, detected by both methods is presented in Supplementary Table S3. The majority of the observed events were skipped exons (SE), with only a handful of alternative 5′ or 3′ splice sites detected (Fig. 3a, Supplementary Tables S3 and S5), suggesting that loss of QKI may result in significant changes at the proteome level. Importantly, most SE events corresponded to skipped cassette exons (Fig. 3a). Among AS events identified by DEXSeq only, approximately half affected first or last exons (Fig. 3a). Since alternative promoter or terminator usage is generally not regulated via the splicing machinery, we excluded them from further analyses and concentrated on cassette exons only. We showed previously that the QKI isoforms bind optimally to QKI response elements (QREs) with a sequence of ACUAAY (N1-20) UAAY^17^. Therefore, we searched for the core sequence ACUAA as a minimal sequence and whether it is enriched upstream and/or downstream of the skipped cassette exons (SCE) events (Figs 3b,c and S2). More than 40% of SCE events from the stringent list had the exact sequence motif ACUAA in the neighbouring regions (Supplementary Table S7). In particular, this was the case for almost 50% of SCE events with lower inclusion level in *QKI* ^*FL*/*FL*;*Olig2*-*Cre*^ mice. In both cases, this represents statistically significant enrichment (p-value < 0.001) over the distribution of the ACUAA motif frequency in a random set of control sequences (Figs 3b and S2b). Furthermore, over 30% of the complete set of 842 detected SCE events had the sequence motif in neighbouring sequences (Supplementary Table S8), again representing a significant enrichment over the control distribution (Figs 3c and S2c,d). In addition, we determined whether the motif was present upstream or downstream of the AS exon. Similar to previous reports^3^, we observed that the QRE core sequence tends to be located more often upstream of ASEs that are repressed by QKI and downstream of ASEs that are activated by QKI (Supplementary Tables S7, S8 and Supplementary Fig. 2g).

**Figure 4:**
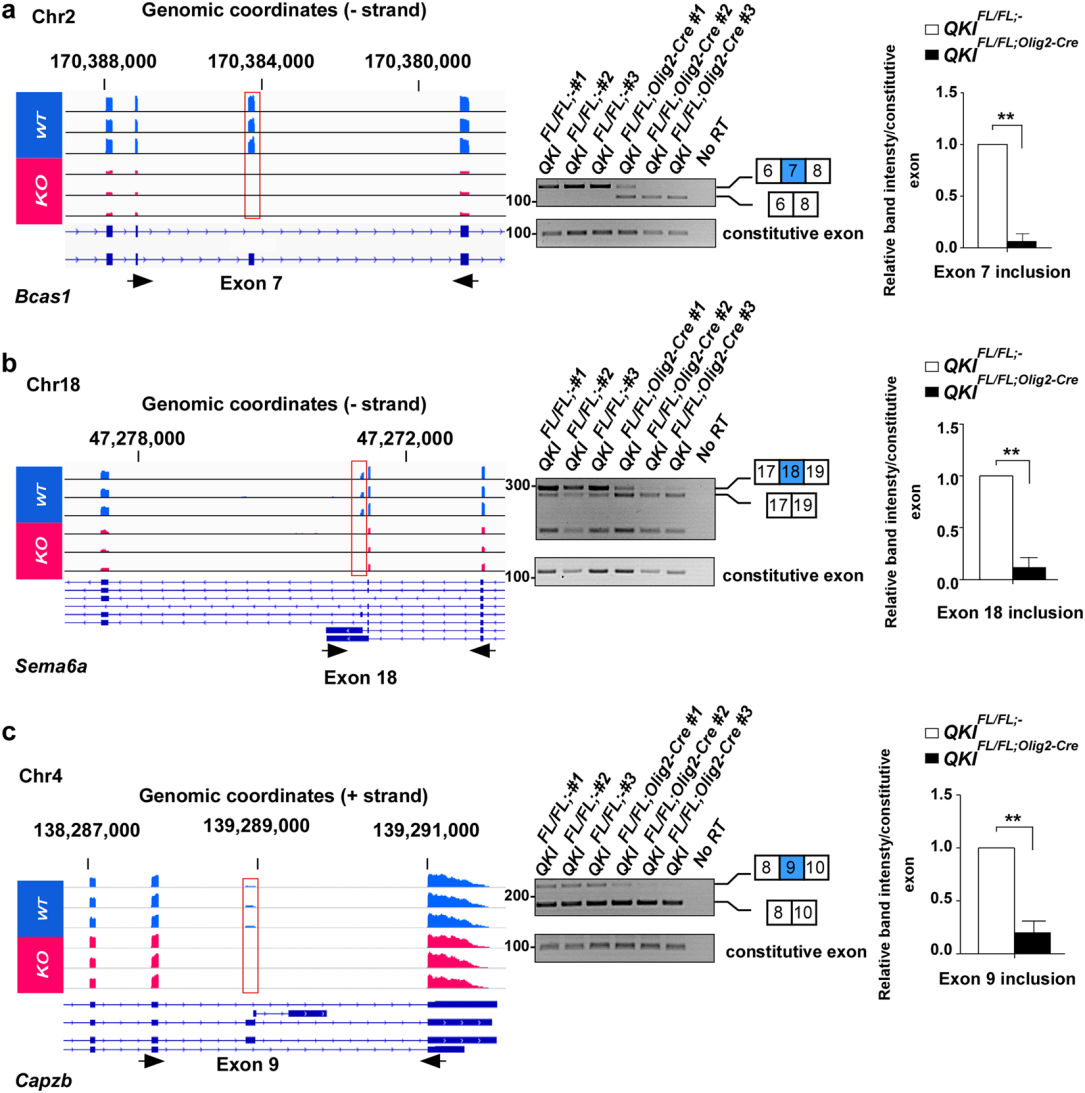
Alternative splicing event in Bcas1, Sema6a and Capzb in *QKI* ^*FL*/*FL*;*Olig2*-*Cre*^ and *QKI* ^*FL*/*FL*;-^ mice. (**a**,**b**,**c**) Left: IGV views of the exon skipping events in *Bcas1*, *Sema6a* and *Capzb*. All samples are represented on the same scale for each screenshot. Black arrows represent the primers used for validation by RT-PCR. Right: Validation of exon skipping events by semi-quantitative PCR in n = 3 mice per genotype. The constitutive exon is present in all isoforms of the gene and is not alternatively spliced. The pixel densities of PCR bands from 3 independent mice brain RNA samples were quantified using ImageJ software, normalized over wild-type mice and represented as mean ± SEM (Student *t*-test, **p < 0.001).

We performed GO analysis on the stringent list of candidates. Interestingly, the top enriched GO terms for biological processes included regulation of gliogenesis and myelination with myelin structural constituents and paranodal junction being one of the top molecular functions and cellular components, respectively (Fig. 3d). Moreover, many of the top AS candidates are associated with neurological disease(s) and/or are involved in neurite outgrowth, neurogenesis, oligodendrocyte differentiation and myelination (Table 1). In some cases, we found almost complete exon skipping in *QKI* ^*FL*/*FL*;*Olig2*-*Cre*^, such as for exon 7 of *Bcas1*, exon 18 of *Sema6a* and exon 9 of *Capzb* (Fig. 4a,b,c, left panels, sashimi plots are shown in Supplementary Fig. S4a,b,c), which we have validated by semi-quantitative PCR showing complete exon exclusion in the *QKI* ^*FL*/*FL*;*Olig2*-*Cre*^ mice (Fig. 4a,b,c, middle panels). We have also quantified the exon inclusion levels normalized over a constitutive exon present in all isoforms for each of those three genes and a significant reduction was observed (p < 0.001. Fig. 4a,b,c).

**Table 1:** A large number of novel AS events observed in *QKI* ^*FL*/*FL*;*Olig2*-*Cre*^ mice are located in genes associated with neurological diseases and/or involved in important brain function, with the top 15 represented in this table.

Gene Symbol	Gene Description	Splicing inclusion change in *QKI* ^*FL*/*FL*;*Olig2*-*Cre*^	Event Type	Associated neurological diseases/brain function	Reference (PMID)
Vcan	versican	inclusion	SE	Neurite outgrowth	24955366
Gpm6b	glycoprotein m6b	exclusion	A3SS	Myelination	23322581
Ptprz1	protein tyrosine phosphatase, receptor type Z, polypeptide 1	inclusion/exclusion	SE	Oligodendrocyte differentiation and myelination	23144976
Mag	myelin-associated glycoprotein	inclusion	SE	Associated with demyelinating leukodystrophy	26179919
Sema6a	semaphorin 6A	exclusion	SE	Oligodendrocyte differentiation and myelination	22777942
Ndrg2	N-myc downstream regulated gene 2	inclusion	SE	Mainly expressed in astrocytes, regulates neurogenesis, associated with neurodegeneration	26341979
Ank3	ankyrin 3, epithelial	exclusion	SE	Genetic risk factor for psychiatric disorders; cytoskeletal scaffolding and synaptic protein	25374361
Daam2	dishevelled associated activator of morphogenesis 2	inclusion	SE	Suppresses oligodendrocyte differentiation during development, elevated in Guillain-Barré syndrome	25754822 26293489
Epb4.1l2	erythrocyte protein band 4.1-like 2	inclusion	SE	Organization of internodes in peripheral myelin	22291039
Map4k4	mitogen-activated protein kinase kinase kinase kinase 4	inclusion	SE	Neurite outgrowth and retraction	18007665
Myo6	myosin VI	exclusion	SE	Hippocampal synaptic transmission	16819522
Map4	microtubule-associated protein 4	inclusion	SE	Microtubule-stabilizing activity in oligodendrocytes and astrocytes	15642108
Bcas1	breast carcinoma-amplified sequence 1	exclusion	SE	Among most abundant transcripts in myelin	27173133
Fgfr3	fibroblast growth factor receptor 3	inclusion	RI	Involved in neurogenesis, associated with hypochondroplasia, medial temporal lobe dysgenesis and focal epilepsy	26180198 24630288
Map2	microtubule-associated protein 2	exclusion	SE	Microtubule-stabilizing activity and regulation of microtubule networks in dendrites	15642108

Abbreviations: SE: skipped exon, A5SS: alternative 5′ splice site, A3SS: alternative 3′ splice site, RI: retained intron.

### Quaking regulates the alternative splicing of its pre-mRNA

**Figure 5:**
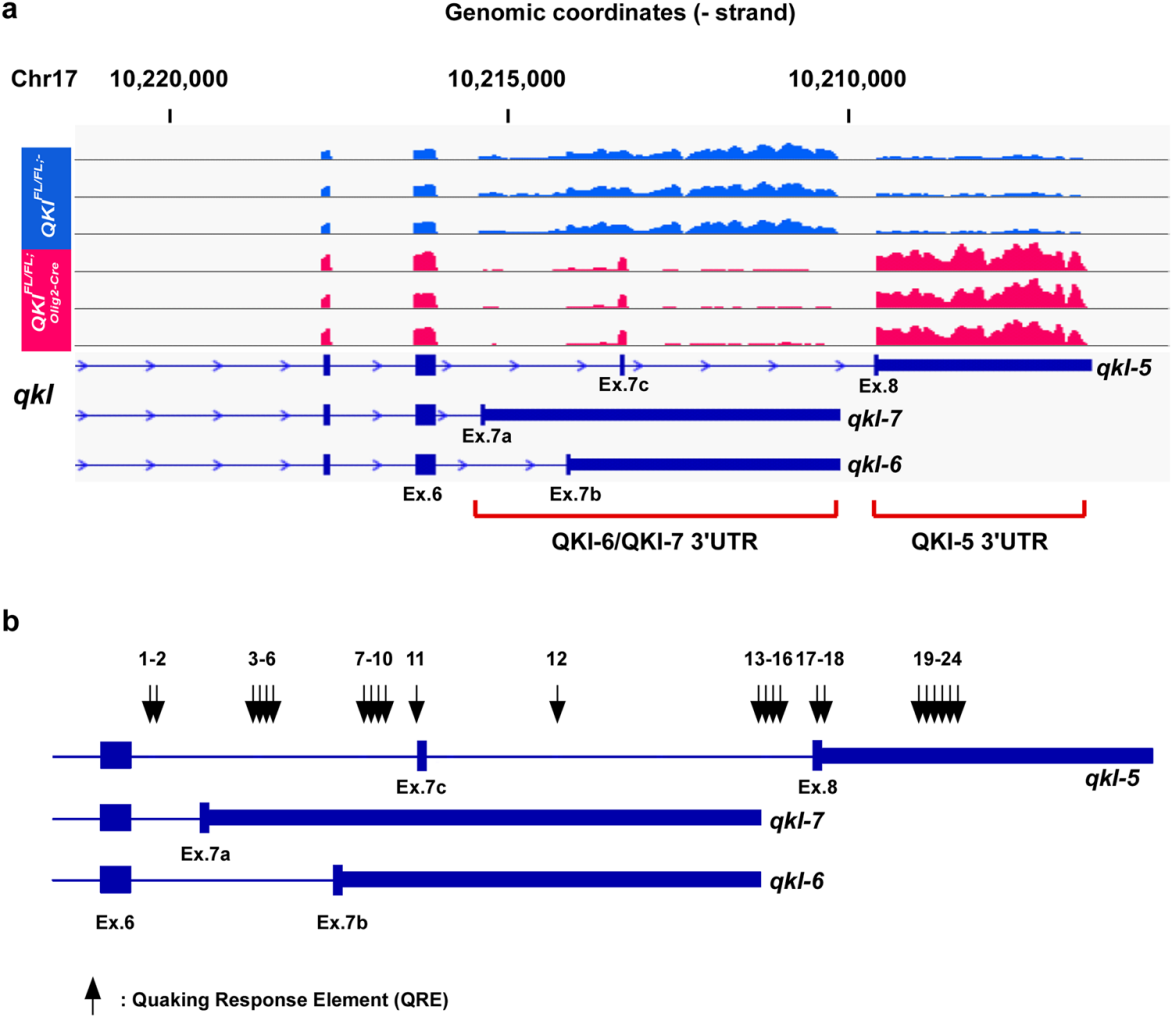
Alterative splicing patterns of *qkI* in *QKI* ^*FL*/*FL*;*Olig2*-*Cre*^ *and QKI* ^*FL*/*FL*;-^ mice. (**a**) IGV view of the alternative splicing events in *qkI*. All samples are represented on the same scale for each screenshot. (**b**) A schematic of the 3′-UTR of mouse *qkI* gene showing the location of Quaking Response Elements (QREs) identified. QREs are either a perfect match to consensus QRE or differ by one mismatch.

**Figure 6:**
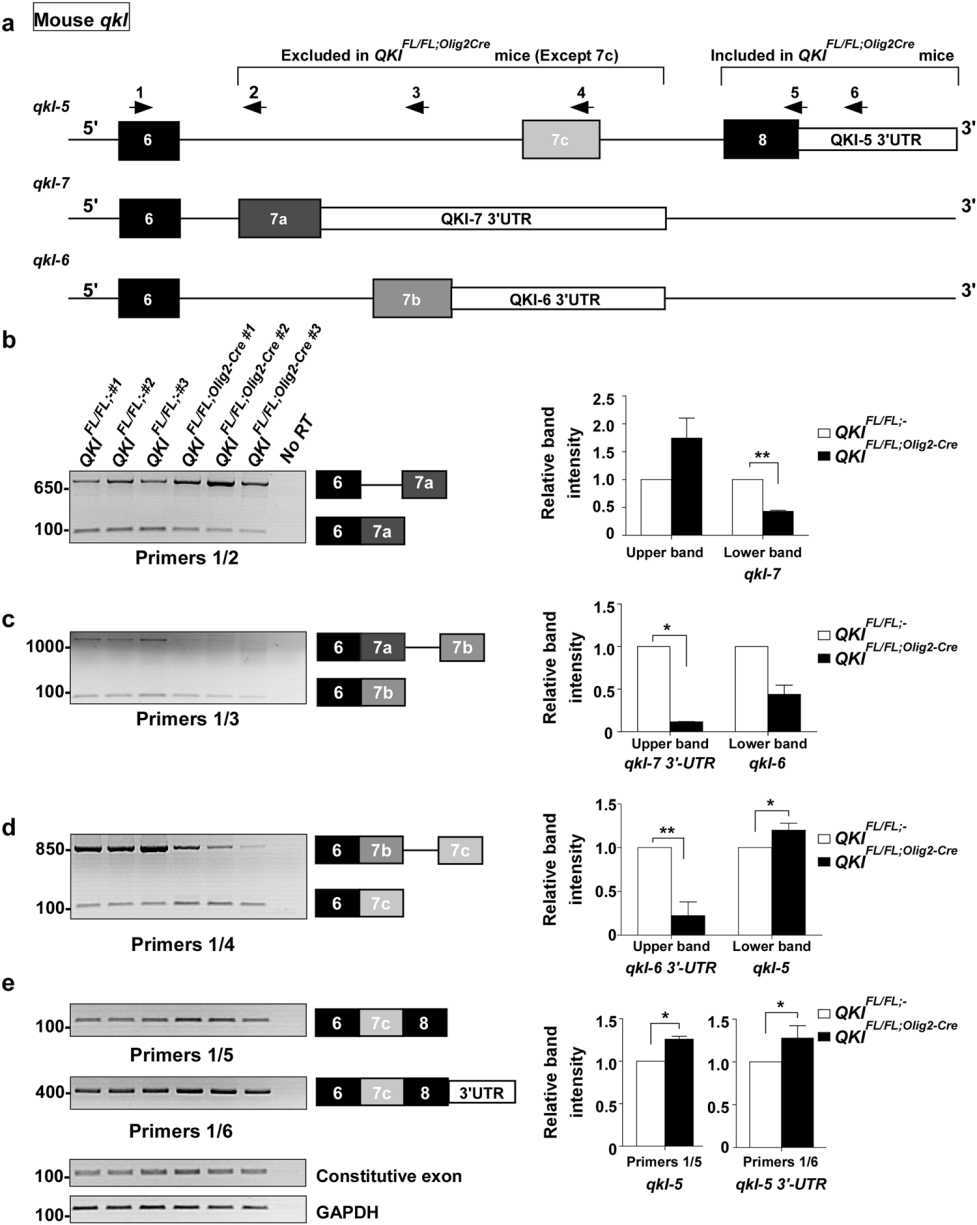
QKI proteins regulate self-splicing in mice. (**a**) A schematic of the 3′-UTR of mouse *qkI* gene. Arrows indicate location of primers used for splicing analysis. Primer 1 is in exon 6 that is common to all QKI isoforms. (**b**) (Left) RT-PCR analysis using primers #1 and #2 from n = 3 mice brain RNA/genotype. The lower band corresponds to *qkI-7* isoform (exon 6; 7a) whereas the upper band corresponds to the unspliced pre-mRNA. (Right) The pixel densities of PCR bands from 3 independent mice brain RNA samples were quantified using ImageJ software, normalized over constitutive exon, and then normalized over wild-type mice represented as mean ± SEM (Student *t*-test, **p < 0.001). (**c**) (Left) RT-PCR analysis using primers #1 and #3 from n = 3 mice brain RNA/genotype. The lower band corresponds to *qkI-6* isoform (exon 6; 7b) whereas the upper band corresponds to *qkI-7* 3′-UTR. (Right) The pixel densities of PCR bands from 3 independent mice brain RNA samples were quantified using ImageJ software, normalized over constitutive exon and then normalized over wild-type mice and represented as mean ± SEM (Student *t*-test, *p < 0.05). (**d**) (Left) RT-PCR analysis using primers #1 and #4 from n = 3 mice brain RNA/genotype. The lower band corresponds to *qkI-5* isoform (exon 6; 7c) whereas the upper band corresponds to *qkI-6* 3′-UTR. (Right) The pixel densities of PCR bands from 3 independent mice brain RNA samples were quantified using ImageJ software, normalized over constitutive exon and then normalized over wild-type mice and represented as mean ± SEM (Student *t*-test, *p < 0.05, **p > 0.01). (**e**) (Left) RT-PCR analysis using primers #1 and #5 (*qkI-5* isoform: exon 6; 7c; exon8), primers #1 and #6 (*qkI-5* isoform: exon 6; 7c; exon8; 3′-UTR), constitutive *qkI* exon 1, and GAPDH from n = 3 mice brain RNA/genotype. (Right) The pixel densities of PCR bands from 3 independent mice brain RNA samples were quantified using ImageJ software, normalized over constitutive exon and then normalized over wild-type mice and represented as mean ± SEM (Student *t*-test, *p < 0.05).

**Figure 7:**
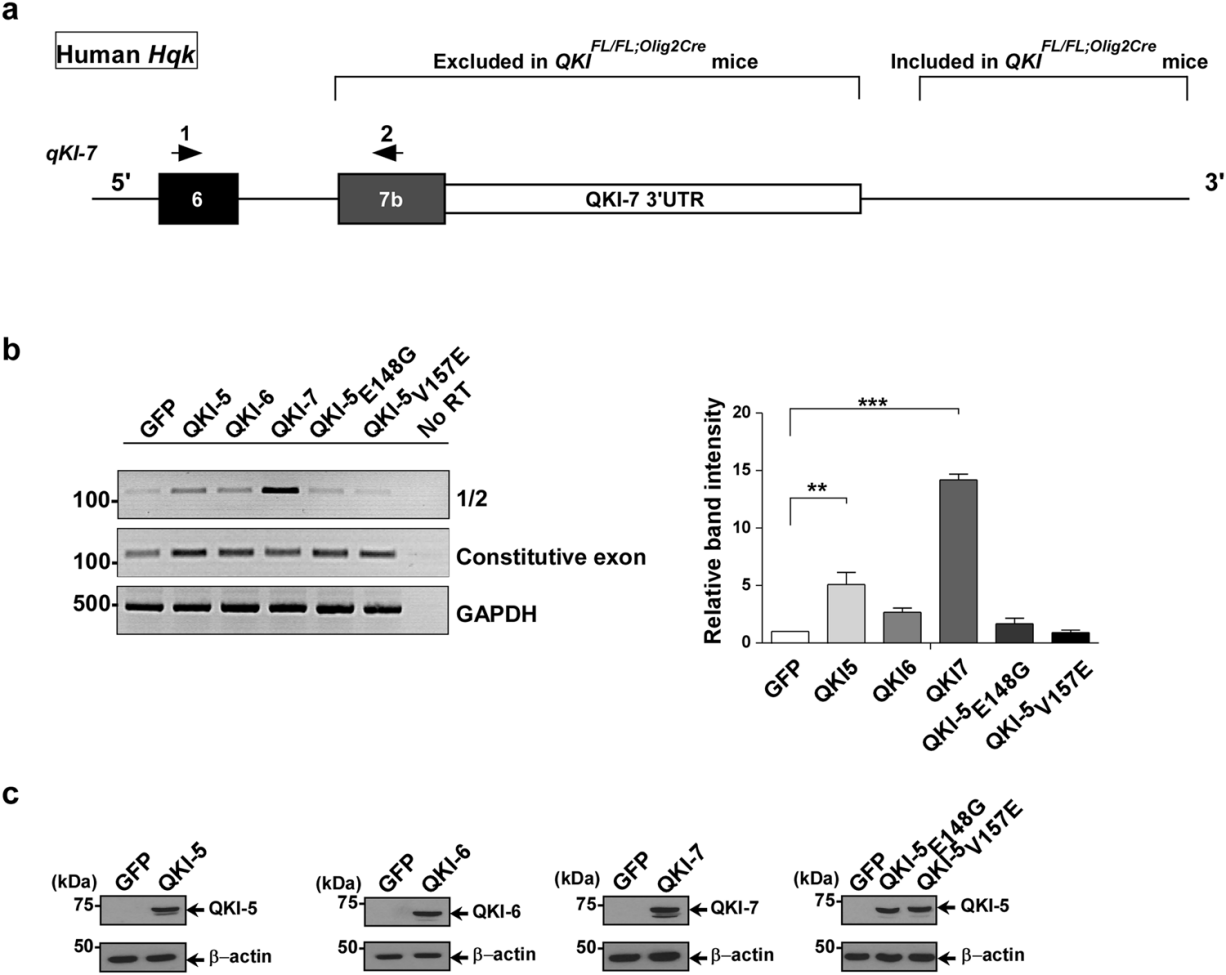
QKI-5 regulates self-splicing *in vitro* in human cells. (**a**) A schematic of the 3′-UTR of human *Hqk* gene. Arrows indicate location of primers used for RT-PCR/splicing analysis. (**b**) (Left) HEK293T cells were transfected with expression vectors encoding green fluorescent protein (GFP) alone or expression vectors for GFP-QKI-5, -QKI-6, -QKI-7, -QKI-5:V157E or -QKI-5:E148G. Total RNA was isolated 72 h later and analyzed by RT-PCR (n = 3). The pixel densities of PCR bands from 3 independent transfections were quantified using ImageJ software, normalized over *Hqk* constitutive exon 1 and represented as mean ± SEM (One-way ANOVA, **p < 0.01, ***p < 0.001). (**c**) Protein expression of transfected plasmids in HEK293T cells is determined by immunoblotting using the indicated QKI antibodies or β-actin antibody for equivalent loading. The molecular mass markers are shown on the left in kDa and the migration of the GFP-QKI isoforms indicated on the right.

Remarkably, among the most significant events detected were exons of *qkI* itself, indicating a switch of isoform usage in *QKI* ^*FL*/*FL*;*Olig2*-*Cre*^ mice. The alternative splicing of exons 7 and 8 in the 3′ UTR of the *qkI* gene generates the different QKI isoforms. In fact, exons 7c and 8 as well as the 3′ UTR of the *qkI-5* isoform were significantly upregulated, while exons 7a, 7b and the 3′ UTRs of *qkI-6* and *qkI-7* isoforms were significantly downregulated in *QKI* ^*FL*/*FL*;*Olig2*-*Cre*^ mice compared to *QKI* ^*FL*/*FL*;-^ mice (Fig. 5a, sashimi plots are shown in Supplementary Fig. S4d). We also searched the *qkI* gene for QREs and we were able to identify many that mapped in intronic or exonic sequences. We identified 24 QREs present in the 3′ alternatively spliced region of *qkI* that harbour either a perfect QRE or differ by 1 mismatch (Fig. 5b and Supplementary Table S9). We next designed primer pairs aimed at amplifying the different *qkI* isoforms with their locations and expected products shown in Supplementary Table S10. We validated the alternative splicing of *qkI* pre-mRNA by RT-PCR using RNA from brains of three mice each for *QKI* ^*FL*/*FL*;-^ and *QKI* ^*FL*/*FL*;*Olig2*-*Cre*^. Since exon 6 of *qkI* mRNA is common to all the isoforms, we designed a primer (Fig. 6a; primer #1) that is located in this exon and performed PCR with primers that map to either *qkI-5* (Fig. 6a; primer#4), *qkI-6* (Fig. 6a; primer#3) or *qkI-7* (Fig. 6a; primer#2) specific exon 7 as well as in *qkI-5* specific exon 8 (Fig. 6a; primer#5) and 3′-UTR (Fig. 6a; primer#6). *qkI-5*, *qkI-6* and *qkI-7* share overlapping sequences in the 3′-end and therefore these primers amplify regions in each of these transcripts with the size of the PCR product indicating which *qkI* isoform is being amplified. In *QKI* ^*FL*/*FL*;*Olig2*-*Cre*^ mice, we observed a decrease in the *qkI-7* isoform as evident by the lower PCR product obtained from primers #1 and #2 that corresponds to exon 6; exon 7a (Fig. 6b). The upper band in Fig. 6b corresponds to a product if the intron was retained (unspliced pre-mRNA) and shows a non-significant increase in *QKI* ^*FL*/*FL*;*Olig2*-*Cre*^ mice. Primers #1 and #3 show a decrease in the *qkI-6* isoform (the lower PCR product in Fig. 6c that corresponds to exon 6; exon 7b). The upper band corresponds to the 3′-UTR of *qkI-7* which shows a significant decrease in the *QKI* ^*FL*/*FL*;*Olig2*-*Cre*^ mice. Primers #1 and #4 show a significant increase in *qkI-5* isoform (Fig. 6d, lower band, exon 6; exon 7c) with a significant decrease in the 3′-UTR of *qkI-6* (Fig. 6d, upper band). A significant increase in *qkI-5* isoform is shown using primers #1 and #5 (Fig. 6e, exon 6; exon 7c; exon 8) as well as an increase in the 3′-UTR of *qkI-5* using primers #1 and #6 (exon 6; exon 7c; exon 8; 3′-UTR). We have also quantified the exon inclusion/exclusion levels normalized over a constitutive exon of *qkI* (in exon 1) shown on the right of each semi-quantitative PCR (Fig. 6b,c,d,e). The observed decrease in the inclusion of exons 7a (*qkI-7*) and 7b (*qkI-6*) and the increase in the inclusion of exon 7c of *qkI*-5 were confirmed by RT-qPCR (Supplementary Fig. S3a). The deletion of exon 2 generates a frame-shift resulting in a null allele and therefore a complete lack of QKI proteins as previously shown^2^. The loss of QKI-6 and QKI-7 specific exons and 3′-UTRs in *QKI* ^*FL*/*FL*;*Olig2*-*Cre*^ brains indicates that expression of the nuclear QKI-5 isoform is required to promote the inclusion of exons 7a (*qkI-7*) and 7b (*qkI-6*) as well as their 3′-UTRs. To confirm this, we transfected plasmids expressing green fluorescent protein (GFP) alone or GFP-QKI-5, -QKI-6, -QKI-7, mutated versions of QKI-5 that either cannot dimerize (GFP-QKI-5:E148G), or a version that cannot bind RNA (GFP-QKI-5:V157E) in HEK293T cells and assessed the inclusion of exon 7b of human QKI-7 by RT-PCR. Note, in humans the qkI-7 exon is 7b and not 7a as in mice (Fig. 7a). We observed an increase in the *Hqk-7* isoform (exon 6; exon 7b) only when GFP-QKI-5 was transfected, but not when GFP-QKI-6, GFP-QKI-7 or the mutated versions of QKI-5 were transfected (Fig. 7a,b). We have also quantified exon 7b (*Hqk-7*) inclusion levels normalized over a constitutive exon of *qkI* (exon 1), and observed a significant increase in exon 7b inclusion when QKI-5 was transfected (Fig. 7b, right). The increase observed when GFP-QKI-7 was transfected is due to the primer being in exon 7b of the cDNA in pGFP-QKI-7, recognizing the ectopically expressed isoform. Equivalent QKI protein expression was confirmed by immunoblotting (Fig. 7c). These findings show that QKI-5 promotes the inclusion of exon 7b of *Hqk-7* in *cellulo*.

## Discussion

The presence of a QKI response element in >1400 mRNAs suggested that the QKI isoforms regulate many genes^17^. In the present manuscript, we perform a transcriptomic analysis using whole brain RNA from *QKI* ^*FL*/*FL*;-^ and *QKI* ^*FL*/*FL*;*Olig2*-*Cre*^ mice and we identify >1,800 genes that are significantly differentially expressed of which 229 genes show an absolute fold change greater than 2. More than 1400 alternative splicing candidates were identified using two methods, DEXseq and rMATS. Gene ontology analysis of the 229 genes has identified genes involved in glial cell differentiation (OLs), axon ensheathment, and myelination to be the most downregulated. Surprisingly, we identified *qkI* as the top alternatively spliced event by DEXseq and we show that QKI-5 regulates the alternative splicing of the *qkI-6* and *qkI-7* isoforms. Inspection of the *qkI* pre-mRNA reveals the presence of many QREs distributed across the intronic regions of these pre-mRNAs. Our findings define the QKI isoforms as master regulators of RNA metabolism in oligodendrocytes and show that QKI-5 isoform autoregulates alternative splicing of its own gene.

Northern blot analysis shows that the *qkI-5* mRNA is highly expressed during embryogenesis and maintained until approximately P14 at which point its expression declines thereafter. In contrast, the mRNA expression of *qkI-6* and *qkI-7* begins embryonically and peaks at P14 and is maintained into adulthood^7, 25^. Thus our observation in P14 wild type mice (*QKI* ^*FL*/*FL*;-^) that *qkI-6* and *qkI-7* are the main mRNA isoforms observed is expected. What is unexpected is that *qkI-5* mRNA is the major isoform observed at P14 in the brains of *QKI* ^*FL*/*FL*;*Olig2*-*Cre*^ mice. These findings suggest that the complete absence of the QKI proteins favors the splicing of the *qkI-5* isoform. It also suggests that the partial *qkI* promoter deletion observed in the *quaking viable* mice^7^ may decrease the transcriptional output of the *qkI* gene such that the levels of *qkI-5* are insufficient to drive the subsequent pre-mRNA splicing of *qkI-6* and *qkI-7* and their expression. Therefore, it is the *qkI* isoform imbalance that blocks cellular differentiation and these findings support the tumor suppressor function of the QKI proteins^26^, as their deletion or isoform imbalance favors cell proliferation. Furthermore, the haploinsufficient expression of the human *qkI* gene^5^ is enough to block cellular differentiation, as observed with its monocyte to macrophage differentiation defect^4^.

We next compared our mouse alternative splice analysis with two other RNA-seq analyses that were performed in human cells. The *qkI* haploinsufficient patient exhibits intellectual disabilities, hypotonia, seizures, brain anomalies and specific dysmorphic features^5^. She carries a balanced reciprocal translocation (t(5; 6) (q23.1; q26), where a break point in one of her *qkI* alleles causes reduced expression of QKI mRNA and protein^4^. RNA-seq analysis of her monocytes and macrophages compared to her control sibling revealed 1,513 alternative splice events^4^. These spliced events were indeed enriched for QREs next to these regulated exons^4^, suggesting these events are mainly direct targets of the QKI isoforms. Low QKI levels is a prognostic marker for poor survival for patients with NSCLC^26^. The human lung/bronchus epithelial cell line, BEAS-2B, was depleted of the QKI isoforms using shRNAs^26^. RNA-seq was performed and 451 ASE were identified of which 81 were validated by RT-qPCR^26^. Recently, transcriptomic profiling and alternative splicing analysis was performed in wild type versus *qkI*-deficient neural stem cells either in the presence or absence of *Pten* and *Trp53* identifying 388 and 215 genes that are differentially regulated and alternatively spliced, respectively^27^.

Our RNA-seq analysis identified a total of 1438 ASEs (Tables S3 to S5) with 190 events (Supplementary Table S3) that we termed ‘stringent’, as they were identified by both DEXSeq and rMATS. Comparing our stringent list with Zong *et al*.^26^, de Bruin *et al*.^4^ and Shingu *et al*.^27^, we have 96, 93, and 46 common gene names, respectively. If we compare all ASEs from all 4 gene sets, we can assemble a common signature of 4 genes in QKI-depleted cells (ADD3, Bin1, MAP4K4 and WDFY3). Interestingly, this list of 4 gene names spans mouse, human, oligodendrocytes, neural stem cells, monocyte/macrophages and lung epithelial cells. The other alternative splice targets are, therefore, more tissue- or cell-specific. Other genes commonly identified in three out of four gene sets include HP1BP3, MAP3K7, SBF1, SPAG9 and VLDLR.

In sum, we define the QKI RNA binding proteins as master regulators of RNA metabolism driving oligodendrocyte differentiation. Our RNA-seq analysis defines QKI as regulators of alternative splicing and herein we define many spliced targets including self-regulation.

## Materials and Methods

### Isolation of brain RNA

All mouse procedures were performed in accordance with McGill University guidelines, which are set by the Canadian Council on Animal Care. Experimental protocols involving animal use were approved by the McGill University Animal Care Committee. *QKI* ^*FL*/*FL*;*Olig2*-*Cre*^ and *QKI* ^*FL*/*FL*;-^ mice were generated as described^2^. Exon 2 of the *qkI* gene is flanked with loxP sites and the recombination with Cre results in a null. Total RNA was extracted from brains of *QKI* ^*FL*/*FL*;-^ and *QKI* ^*FL*/*FL*;*Olig2*-*Cre*^ P14 mice (n = 3 for each genotype) with appropriate amounts of TRIzol® reagent according to the manufacturer’s instructions (Invitrogen). 30 µg of RNA were DNase I (Promega) treated for 30 mins at 37 °C.

### RNA-Sequencing analysis

Total RNA from *QKI* ^*FL*/*FL*;-^ and *QKI* ^*FL*/*FL*;*Olig2*-*Cre*^ brains (n = 3 females for each genotype) were purified using polyA selection and converted into cDNA by Illumina TruSeq and sequenced on a HiSeq 2000 (Illumina) with paired ends 100 bp in length (Genome Quebec, Canada) at an average of 133 million reads/sample. Illumina sequencing adapters were removed from the reads, and all reads were required to have a length of at least 32 bp. Sequencing runs were processed with Illumina CASAVA software. Trimmomatic v0.32^28^ was used to trim reads, including removal of low-quality bases at the end of reads (phred33 < 30), clipping of the first three bases and clipping of Illumina adaptor sequences using the palindrome mode. We performed quality trimming with a sliding window, cutting once the average quality of a window of four bases fell below 30. We discarded reads shorter than 32 base pairs after trimming. Trimmed reads were aligned to the reference genome mm 10 using STAR v2.3.0e^29^. Quality control was performed using metrics obtained with FASTQC v0.11.2, SAMtools^30^, BEDtools^31^ and custom scripts. This showed a good proportion of high quality reads and mapping rates higher than 90% in all samples. Bigwig tracks were produced with custom scripts, using BEDtools and UCSC tools. Data were visualized using the Integrative Genomics Viewer (IGV)^32^.

### Gene expression analysis

Expression levels were estimated by featureCounts v1.5.0 using exonic reads^33^ and normalized using DESeq2^34^. Global gene expression changes were assessed by unsupervised hierarchical clustering of samples and PCA, as previously described^35^. Differential expression analysis between *QKI* ^*FL*/*FL*;-^ and *QKI* ^*FL*/*FL*;*Olig2*-*Cre*^ mice was performed using DESeq2^34^ for all UCSC known genes. Genes were considered differentially expressed if they had an adjusted p-value < 0.05 and a base mean >100. Expressed genes were defined as all genes with the DESeq2 base mean higher than the first expression quartile.

### Alternative splicing analysis

We used rMATS v3.2.2 to identify alternative splicing (AS) events^24^. Since rMATS requires reads of equal lengths, we performed the analysis on untrimmed reads after verifying that the adaptor content was low in the FASTQC reports. We ran rMATS with standard parameters and analyzed both outputs: AS events identified using reads mapping to splice junctions and reads on target (JC + ROT), and AS events identified using reads mapping to splice junctions only (JC). The list of putative AS events was obtained by including all events with an adjusted p-value (FDR) <0.05 in either JC + ROT or JC. We also computed differential exon usage with DEXSeq v1.12.2^23^. To generate counts compatible with DEXSeq, we used featureCounts to count reads mapping to all annotated exons in Ensembl GRCm38.84 and converted them to the standard DEXSeq input format using scripts available from Vivek Bhardwaj (Subread_to_DEXSeq, https://github.com/vivekbhr/Subread_to_DEXSeq). Differential exon usage analysis between WT and *QKI* KO mice was performed using standard DEXSeq parameters. Exons with a minimum length of 15 nucleotides and with an adjusted p-value > 0.05 were considered statistically significant.

To obtain a reduced set of stringent, robust ASE candidates, we combined predictions from DEXSeq and rMATS, since the underlying methods and the type of information (read counts, splice junction reads, etc) used by each approach are quite dissimilar. Significant events called both by rMATS (p-value < 0.05) and by DEXSeq (adjusted p-value < 0.05) were intersected based on genomic location. For this, we first merged the two rMATS outputs (JC + ROT and JC) so that each AS event was represented by one entry. We then used the BEDTools intersect function to identify the genomic regions of overlap between the results from rMATS and DEXSeq, keeping the information from rMATS (Table S3). Since a number of exons are present as multiple entries in the rMATS outputs (for each isoform they belong to), we then intersected (BEDTools intersect) the results from rMATS and DEXSeq with the GENCODE VM11 annotation to identify all unique AS exons. AS events that were identified by rMATS and DEXSeq but that were not annotated in GENCODE VM11 were then added to the list. Exons with identical genomic coordinates were merged and counted only once. This approach allowed us to count the number of unique AS events without duplicates. To obtain a more comprehensive list of AS exons, we generated the union of the DEXSeq (adjusted p-value < 0.05) and rMATS (adjusted p-value < 0.05). For both the stringent and the comprehensive lists, we then used the GENCODE annotation to distinguish SE events corresponding to cassette exons compared to first or last exons. Exons were considered to be “cassette exons” if they were not the first or last exon in any of the gene isoforms or “mixed exons” if they were not the first or last exon in at least one isoform. SE events identified by rMATS that were not included in the GENCODE annotation were counted as “mixed” exons. This annotation is indicated in the last columns of Tables S3 and S5. Exons that were first or last exons in all isoforms were excluded from further analyses.

### Gene Ontology

GO term enrichment analysis was performed GOrilla^36^. The lists of differentially expressed genes and genes containing AS events were compared to a background list of expressed genes, consisting of all expressed genes in the complete dataset (see Gene expression analysis above). For differentially expressed genes, upregulated and downregulated genes with a base mean higher than 100 and an absolute fold change greater than 2 were used for the analysis. For AS, we used the genes in which stringent AS events (detected by DEXSeq and rMATS, see above) were observed.

### Motif enrichment analysis

We searched for the motif ACUAA in windows of 200 nucleotides neighbouring each side of the identified SE events corresponding to “cassette” or “mixed” exons (see Alternative splicing analysis above). To verify enrichment of this motif in SEs and compute empirical p-values, we generated a set of control exons (n = 88,217) that met the following criteria: “cassette” or “mixed” exons, good coverage at the exon and gene level (coverage >lowest quartile of exon or gene coverage normalized by exon or gene length), not alternatively spliced (DEXSeq adjusted p-value > 0.99 and rMATS p-value < 0.05) and with a minimum length of 15 nucleotides. To compute empirical p-values, we performed 1,000 iterations, where in each iteration we randomly selected a number of control exons equal to the number of observed AS events, and computed the frequency of occurrence of the ACUAA motif within neighbouring windows of 200nt in these controls exons. Motif analyses were performed successively for all SEs, SEs with higher inclusion (QKI repressed) in *QKI* ^*FL*/*FL*;*Olig2*-*Cre*^ mice and SEs with higher exclusion in *QKI* ^*FL*/*FL*;*Olig2*-*Cre*^ mice (QKI activated). To determine the position of the core motif relative to the exon, we used the FIMO tool^37^ (MEME suite) and searched for exact matches to ACUAA.

### Transfections

HEK293T cells were plated in 6-well plates and transfected with 2 µg of the indicated GFP expression vectors encoding QKI-5, QKI-6, QKI-7, QKI5:E148G and QKI5:V157E. The common forward primer for RT-PCR detection of *qkI* alternative splicing was in exon 6 and is the following: 5′-CCT CAC CCA ACT GCT GCA ATA G-3′. The reverse primer to detect exon 7 of QKI-7 inclusion is: 5′-CAT GAC TGG CAT TTC AAT CC-3′. Human constitutive exon: forward, 5′-GTC GGG GAA ATG GAA ACG AAG-3′ and reverse, 5′-GGT TGA AGA TCC CGC AGA AG-3′. Human GAPDH forward: 5′-CCG CAT CTT CTT TTG CGT CG-3′, Human GAPDH reverse: 5′-TGG GTG TCG CTG TTG AAG TC-3′.

### Reverse transcript - qPCR

1 µg of RNA was reverse transcribed using oligo(dT) primer and M-MLV reverse transcriptase according to the manufacturer’s protocol (Promega). cDNAs were then amplified by PCR using the following conditions: 95 °C for 30 seconds, 60 °C for 30 seconds, 72 °C for 1 minute and repeated for 30 cycles. The PCR products were then separated by agarose gel electrophoresis. For real-time PCR, primers were designed and efficiency tested according to the MIQE guidelines. Real-time PCR was performed in triplicates with a 1:4 dilution of cDNA using SyBR Green PCR Mastermix (Qiagen, Valencia, CA) on 7500Fast Real-Time PCR System (Applied Biosystems, Foster City, CA). All quantification data were normalized to GAPDH using the ΔΔCt method. All quantification data were normalized to GAPDH using the ΔΔCt method or for the *qkI* isoforms they were normalized to a constant exon region.

### Primers

Mouse 1: 5′-TCC TTG AGT ACC CTA TTG AAC CC-3′; Mouse 2: 5′-GGG CTG AAA TAT CAG GCA TGA C-3′; Mouse 3: 5′-TTA GCC TTT CGT TGG GAA AGC-3′; Mouse 4: 5′-GGT CTG CGG TCA CAA TCC TTT G-3′; Mouse 5: 5′-TAG GTT AGT TGC CGG TGG C-3′; Mouse 6: 5′-GGC CTT CTT ACC GTT CG-3′;. Mouse constitutive exon (exon 1 in all isoforms): forward, 5′-GTC GGG GAA ATG GAA ACG AAG-3′ and reverse, 5′-GGT TGA AGA TCC CGC AGA AG-3′. Mouse GAPDH forward 5′-CCC AGC TTA GGT TCA TCA GG-3′. Mouse GAPDH reverse: 5′-CAA TAC GGC CAA ATC CGT TC-3′. Mouse Bcas1 forward: 5′-GAC ACT GGT TTC ACC TAA CAA G-3′. Mouse Bcas1 reverse: 5′-GTA GCC TTT GTC TTC CCT C-3′. Mouse Bacs1 constitutive forward: 5′-CCC TCT GAT GGC GTT TCT CA-3′. Mouse Bcas1 constitutive reverse: 5′-CCA CGG AGT TTG ACG TTT GG-3′. Mouse Sema6a forward: 5′-GAG ACT GTC ACA ATT CCT TCG-3′. Mouse Sema6a reverse: 5′-GAA CAA GCT GGT CGT TGC-3′. Mouse Sema6a constitutive forward: 5′-CCA AAC ATG CCA ACA TCG C-3′. Mouse Sema6a constitutive reverse: 5′-TGA CAG TCC TGA GGG TAG GG-3′. Mouse Capzb forward: 5′-CTG GTG GAG GAC ATG GAA AAC-3′. Mouse Capzb reverse: 5′-CTG CTG CTT TCT CTT CAA GG-3′. Mouse Capzb constitutive forward: 5′-GTG GTG GAA GTG CAG GAG-3′. Mouse Capzb constitutive reverse: 5′-AGC CGG ATT TGT TGG TTT GC-3′.

## Electronic supplementary material

Supplementary information

Supplementary tables

## References

[1] Darbelli, L. & Richard, S. Emerging functions of the Quaking RNA-binding proteins and link to human diseases. *Wiley Interdiscip Rev RNA* **14**, doi:10.1002/wrna.1344 (2016). 10.1002/wrna.1344 26991871

[2] Darbelli L, Vogel G, Almazan G, Richard S (2016). Quaking regulates neurofascin 155 expression for myelin and axoglial junction maintenance. J Neurosci.

[3] Hall MP (2013). Quaking and PTB control overlapping splicing regulatory networks during muscle cell differentiation. RNA.

[4] de Bruin RG (2016). Quaking promotes monocyte differentiation into pro-atherogenic macrophages by controlling pre-mRNA splicing and gene expression. Nat Commun.

[5] Backx L (2010). Haploinsufficiency of the gene Quaking (QKI) is associated with the 6q terminal deletion syndrome. Am J Hum Genet.

[6] Lek M (2016). Analysis of protein-coding genetic variation in 60,706 humans. Nature.

[7] Ebersole TA, Chen Q, Justice MJ, Artzt K (1996). The quaking gene product necessary in embryogenesis and myelination combines features of RNA binding and signal transduction proteins. Nat Genet.

[8] Wu J, Zhou L, Tonissen K, Tee R, Artzt K (1999). The quaking I-5 protein (QKI-5) has a novel nuclear localization signal and shuttles between the nucleus and the cytoplasm. J Biol Chem.

[9] Pilotte J, Larocque D, Richard S (2001). Nuclear translocation controlled by alternatively spliced isoforms inactivates the QUAKING apoptotic inducer. Genes & Dev..

[10] Larocque D (2002). Nuclear retention of MBP mRNAs in the Quaking viable mice. Neuron.

[11] Li Z, Zhang Y, Li D, Feng Y (2000). Destabilization and mislocalization of the myelin basic protein mRNAs in quaking dysmyelination lacking the Qk1 RNA-binding proteins. J. Neurosci..

[12] Zearfoss NR, Clingman CC, Farley BM, McCoig LM, Ryder SP (2011). Quaking regulates Hnrnpa1 expression through its 3′ UTR in oligodendrocyte precursor cells. PLoS Genet.

[13] Larocque D (2005). Protection of the p27KIP1 mRNA by quaking RNA binding proteins promotes oligodendrocyte differentiation. Nat. Neurosci.

[14] Zhao L, Mandler MD, Yi H, Feng Y (2010). Quaking I controls a unique cytoplasmic pathway that regulates alternative splicing of myelin-associated glycoprotein. Proc Natl Acad Sci USA.

[15] Doukhanine E, Gavino C, Haines JD, Almazan G, Richard S (2010). The QKI-6 RNA binding protein regulates actin-interacting protein-1 mRNA stability during oligodendrocyte differentiation. Mol Biol Cell.

[16] Hardy RJ (1998). QKI expression is regulated during neuron-glial cell fate decisions. J Neurosci Res.

[17] Galarneau A, Richard S (2005). Target RNA motif and target mRNAs of the Quaking STAR protein. Nat Struct Mol Biol.

[18] Hafner M (2010). Transcriptome-wide identification of RNA-binding protein and microRNA target sites by PAR-CLIP. Cell.

[19] Wu JI, Reed RB, Grabowski PJ, Artzt K (2002). Function of quaking in myelination: regulation of alternative splicing. Proc. Natl. Acad. Sci. USA.

[20] van der Veer EP (2013). Quaking, an RNA-binding protein, is a critical regulator of vascular smooth muscle cell phenotype. Circ Res.

[21] Fünfschilling U (2012). Glycolytic oligodendrocytes maintain myelin and long-term axonal integrity. Nature.

[22] Wilkinson G, Dennis D, Schuurmans C (2013). Proneural genes in neocortical development. Neuroscience.

[23] Anders S, Reyes A, Huber W (2012). Detecting differential usage of exons from RNA-seq data. Genome Res.

[24] Shen S (2014). rMATS: robust and flexible detection of differential alternative splicing from replicate RNA-Seq data. Proc Natl Acad Sci USA.

[25] Hardy RJ (1996). Neural cell type-specific expression of QKI proteins is altered in the *quaking* viable mutant mice. J. Neuroscience.

[26] Zong FY (2014). The RNA-binding protein QKI suppresses cancer-associated aberrant splicing. PLoS Genet.

[27] Shingu, T. *et al*. Qki deficiency maintains stemness of glioma stem cells in suboptimal environment by downregulating endolysosomal degradation. *Nat Genet*, doi:10.1038/ng.3711 Nov 14 (2016). 10.1038/ng.3711 PMC5453714 27841882

[28] Bolger AM, Lohse M, Usadel B (2014). Trimmomatic: a flexible trimmer for Illumina sequence data. Bioinformatics.

[29] Dobin A (2013). STAR: ultrafast universal RNA-seq aligner. Bioinformatics.

[30] Li H (2009). The Sequence Alignment/Map format and SAMtools. Bioinformatics.

[31] Quinlan AR, Hall IM (2010). BEDTools: a flexible suite of utilities for comparing genomic features. Bioinformatics.

[32] Thorvaldsdóttir H, Robinson JT, Mesirov JP (2013). Integrative Genomics Viewer (IGV): high-performance genomics data visualization and exploration. Brief Bioinform.

[33] Liao Y, Smyth GK, Shi W (2014). eatureCounts: an efficient general purpose program for assigning sequence reads to genomic features. Bioinformatics.

[34] Love MI, Huber W, Anders S (2014). Moderated estimation of fold change and dispersion for RNA-seq data with DESeq2. Genome Biol.

[35] Binan L (2016). Live single-cell laser tag. Nat Commun.

[36] Eden E, Navon R, Steinfeld I, Lipson D, Yakhini Z (2009). GOrilla: a tool for discovery and visualization of enriched GO terms in ranked gene lists. BMC Bioinformatics.

[37] Grant CE, Bailey TM, Noble WS (2011). “FIMO: Scanning for occurrences of a given motif”. Bioinformatics.

