# Supplementary material for: Transcriptome profiling of mouse brains with qkI-deficient oligodendrocytes reveals major alternative splicing defects including self-splicing
Source: Sci Rep. 2017 Aug 8;7:7554. doi: 10.1038/s41598-017-06211-1 (PMC5548867; doi:10.1038/s41598-017-06211-1)

Supplementary Information

Transcriptome profiling of mouse brains with *qkI*-deficient oligodendrocytes reveals major alternative splicing defects including self-splicing

Lama Darbelli[1#], Karine Choquet[2#], Stéphane Richard[1*], and Claudia L. Kleinman [2*]

Supplementary figure legends:

Supplementary Figure 1. Global effects of loss of QKI in OLs on gene expression. (a) Unsupervised hierarchical clustering (top panel) and Principal Component Analysis (bottom panel) based on expression profiles, using a variable number of most variant genes (from 100 to 10,000). (b) Multiscale bootstrapping of gene expression clustering using the 1,000 most variant genes, performed with the R package pvclust (Bioinformatics 22(12:1540-2), 2006). In red, the approximately unbiased (AU) p-value is represented. With all algorithms, the two groups consistently form distinct, robust clusters, indicating major expression changes associated with genotype. **(c)** RT-qPCR for the indicated genes using P14 brain RNA from n=3 mice/genotype. The results are represented in terms of fold change after normalizing the mRNA levels to a GAPDH and normalized after to wild-type. Each value represents the mean ± SEM.

Supplementary Figure 2. Motif enrichment analysis for QKI activated and QKI repressed SE events. Percentage of sequences with the motif ACUAA in introns neighboring SE events compared to a set of background sequences for the stringent SE candidates called by both methods (a, b) or for all SE candidates (c, d). QKI activated exons correspond to the ones that have lower inclusion level in *QKI*$^{FL/FL;Olig2\text{-}Cre}$ mice, while QKI repressed exons have higher inclusion level in *QKI*$^{FL/FL;Olig2\text{-}Cre}$ mice. The p-value corresponds to the empirical p-value of the enrichment for the set of SE events compared to the control distribution. (e,f) Number of SE that are included or excluded in *QKI*$^{FL/FL;Olig2\text{-}Cre}$ mice compared to *QKI*$^{FL/FL;-}$ mice in the stringent events (e) and all events (f). (g) Density plot of the location of ACUAA sequence relative to exon for QKI

repressed and QKI activated exons. Position of the QRE relative to the exon was computed with FIMO.

Supplementary Figure 3. Alternative splicing of the 3'-UTR of the *qkI* gene.

(a) RT-qPCR using indicated primers. The results are represented in terms of fold change after normalizing the mRNA levels to a constitutive *qkI* exon from n= 3 samples/genotype. Each value represents the mean ± SEM. * denotes $p < 0.05$. *** denotes $p < 0.001$, Student's *t*-test.

Supplementary figure 4. Sashimi plots of alternative splicing events in *Bcas1, Sema6a, Capzb* and *qkI* in $QKI^{FL/FL;Olig2\text{-}Cre}$ *and* $QKI^{FL/FL}$;- mice. Sashimi plots produced with IGV illustrating the exon skipping events in *Bcas1, Sema6a* and *Capzb*, with the number of reads supporting each exon junction. The number of reads in each sample is indicated in grey on the left of the plot. For *Sema6a* and *qkI*, only junctions with more than 5 and 10 supporting reads, respectively, are indicated to maintain legibility.

Supplementary Figure 5. Original PCR gels.

Original PCR gel images from $QKI^{FL/FL}$;- and $QKI^{FL/FL;Olig2\text{-}Cre}$ mice brain RNA.

Supplementary Figure 6. Original PCR gels and western blots.

(a) Original PCR gel images from HEK293T transfected with the indicated plasmids. (b) Original western blots of transfected HEK293T cells with the indicated plasmids blotted for the expression of QKI proteins and β-actin as loading control.

Darbelli L, Choquet K, Richard S, Kleinman C. Supplementary Figure 1

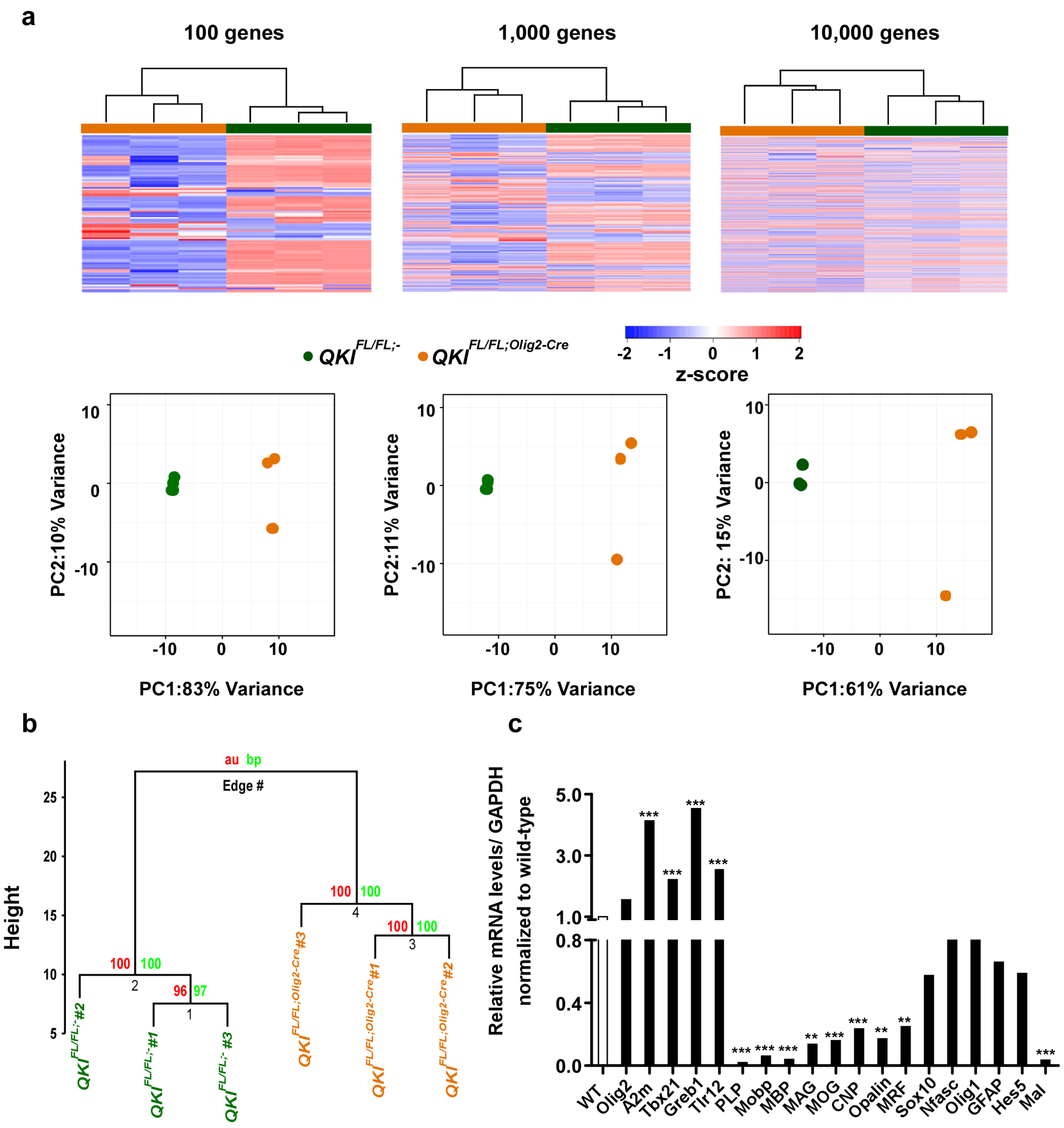

Darbelli L, Choquet K, Richard S, Kleinman C. Supplementary figure 2

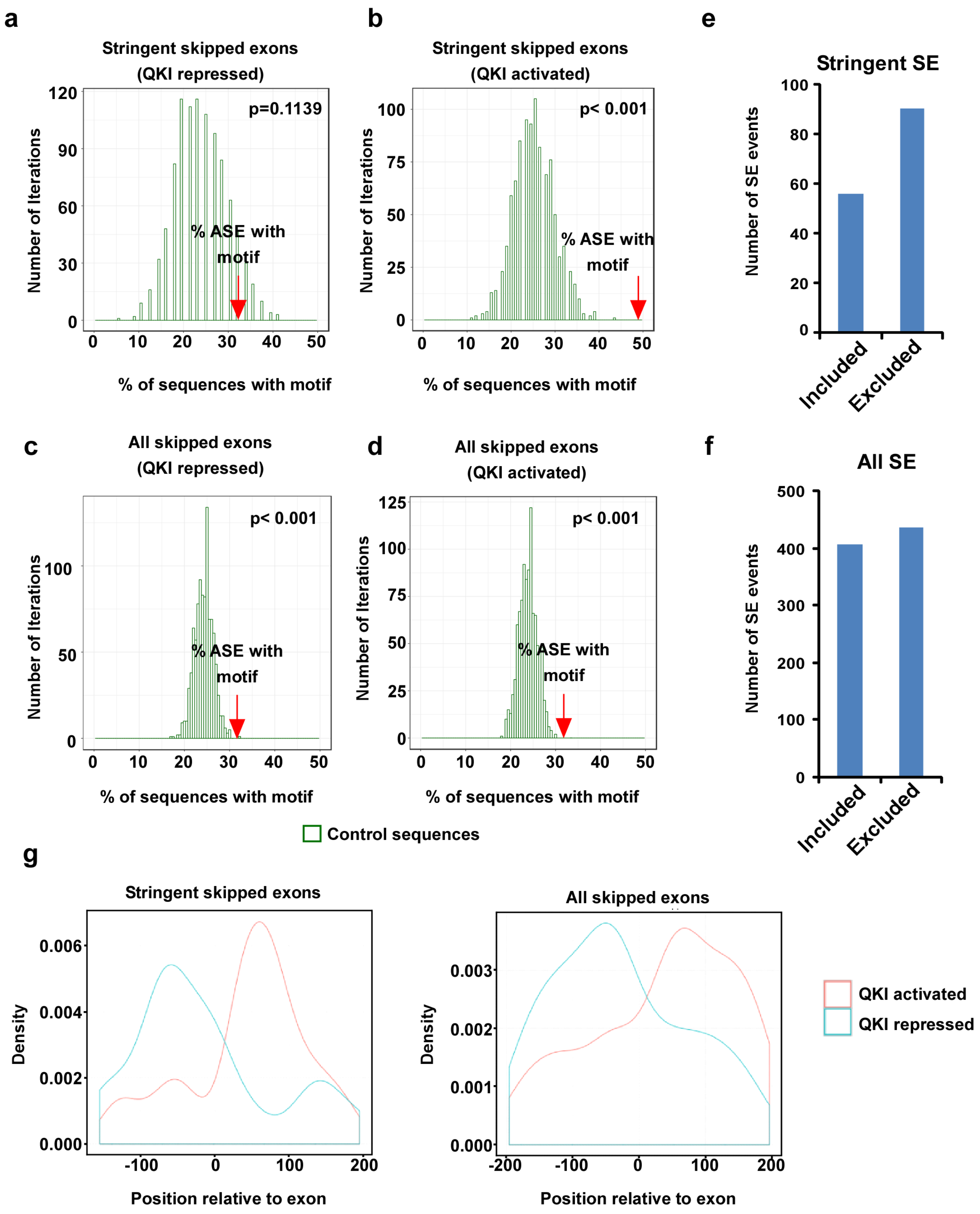

Darbelli L, Choquet K, Richard S, Kleinman C. Supplementary figure 3

a

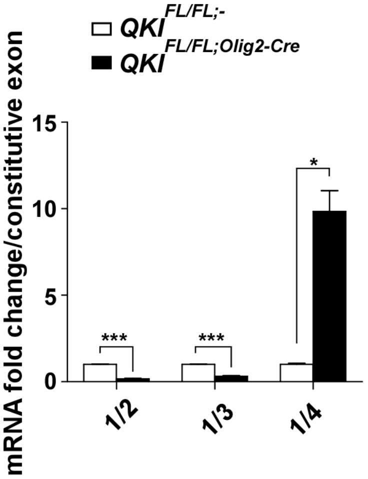

Darbelli L, Choquet K, Richard S, Kleinman C. Supplementary Figure 4

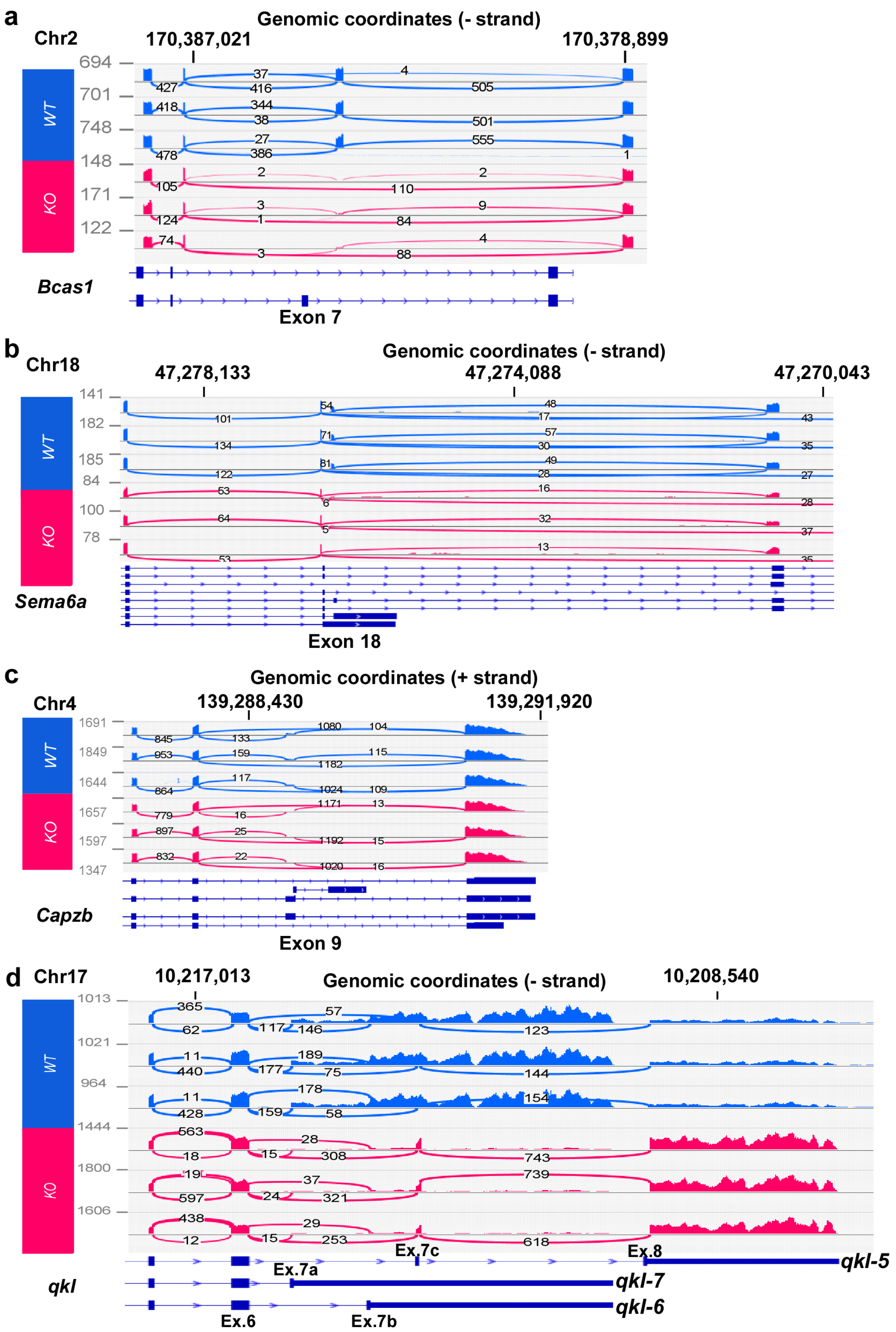

Darbelli L, Choquet K, Richard S, Kleinman C. Supplementary Figure 5

a

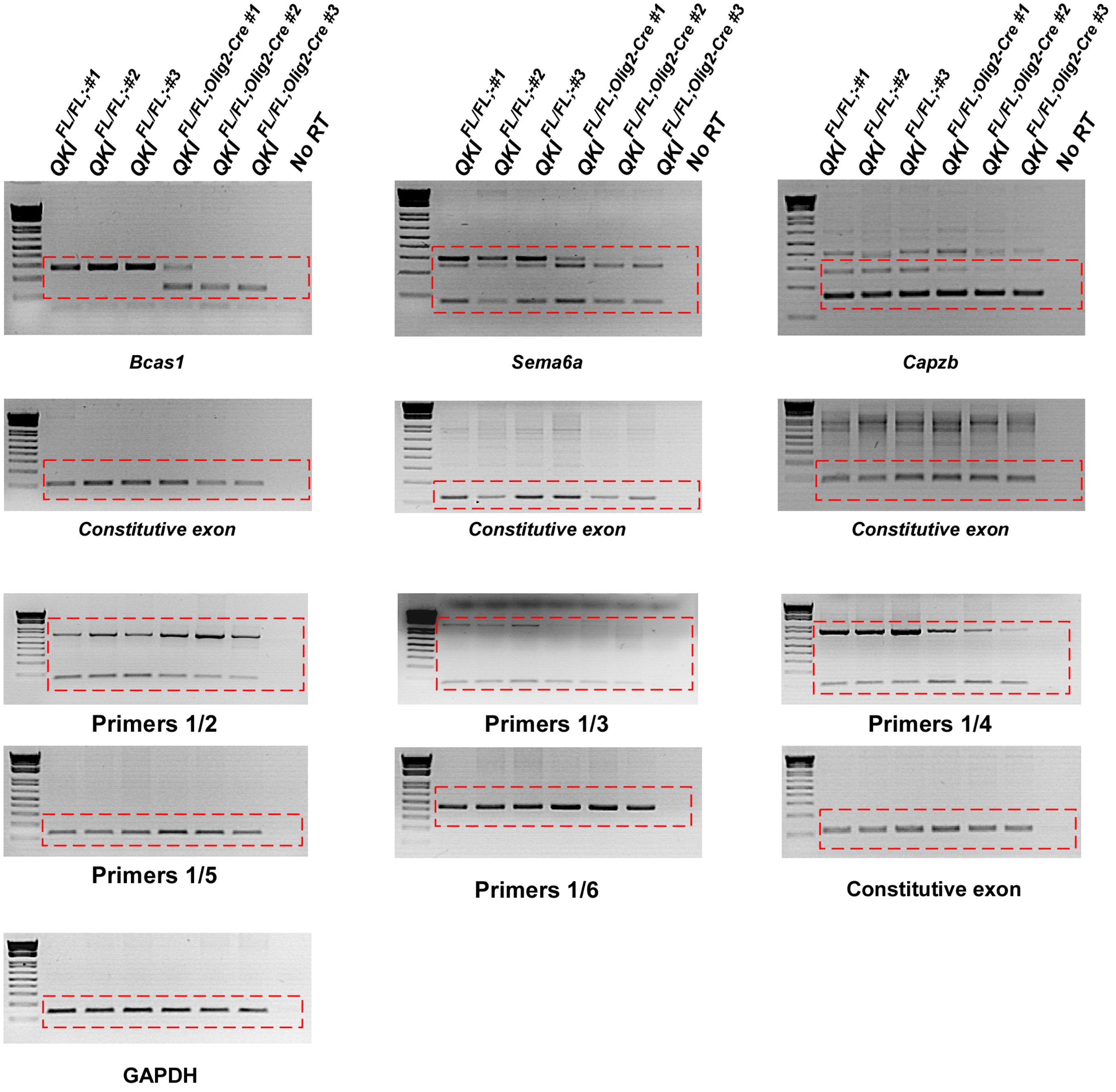

Darbelli L, Choquet K, Richard S, Kleinman C. Supplementary Figure 6

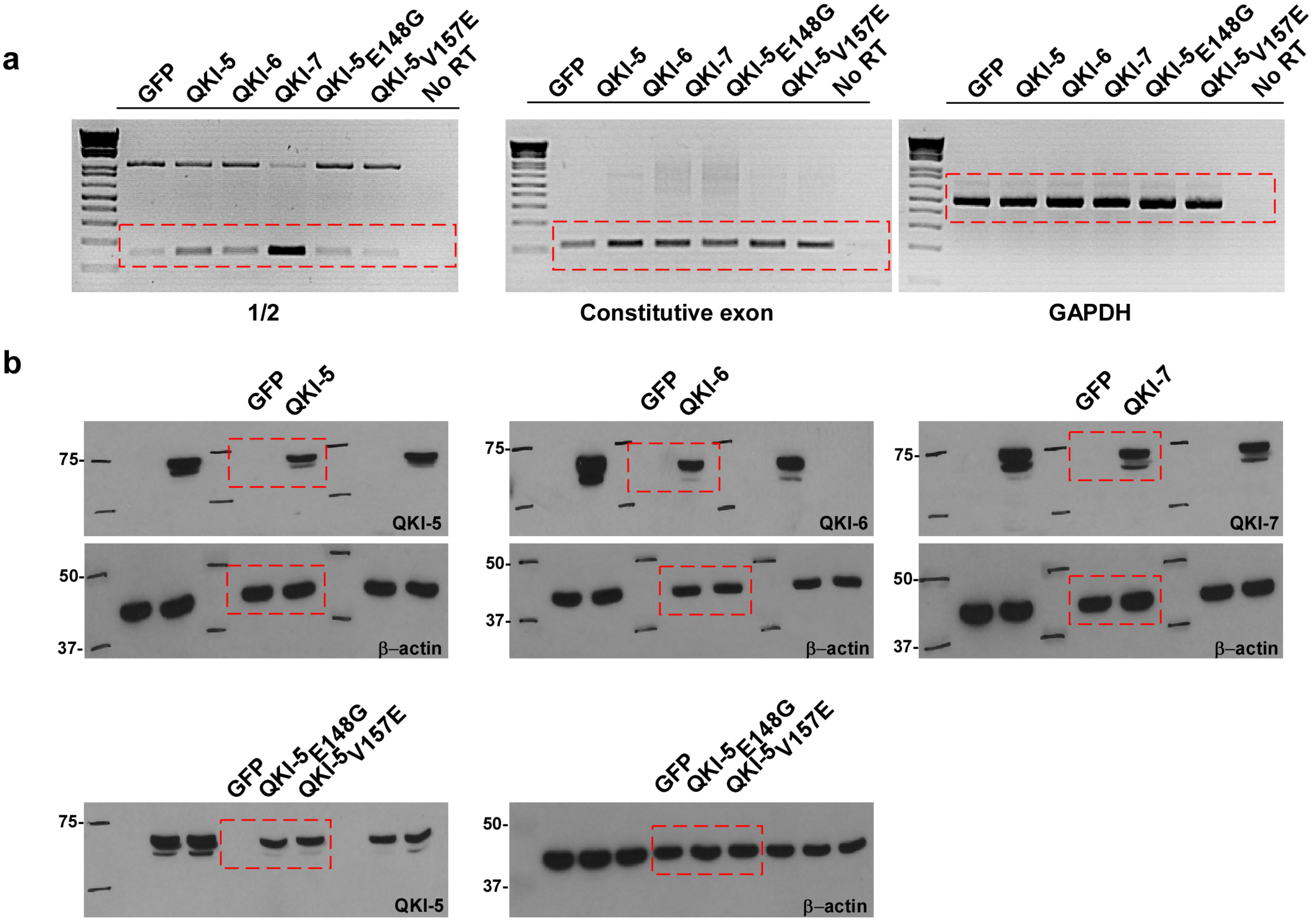

Supplement: Supplementary file 1 — Supplementary information [file 41598_2017_6211_MOESM1_ESM.pdf]
